# Coordinated Sumoylation and Ubiquitination Modulate EGF Induced EGR1 Expression and Stability

**DOI:** 10.1371/journal.pone.0025676

**Published:** 2011-10-05

**Authors:** Arcangela Gabriella Manente, Giulia Pinton, Daniela Tavian, Gerardo Lopez-Rodas, Elisa Brunelli, Laura Moro

**Affiliations:** 1 Dipartimento di Scienze Chimiche, Alimentari, Farmaceutiche e Farmacologiche, University of Piemonte Orientale “A. Avogadro”, Novara, Italy; 2 Department of Psychology, Catholic University of the Sacred Heart, Milan, Italy; 3 Department of Biochemistry, University of Valencia, Valencia, Spain; Université Paris-Diderot, France

## Abstract

**Background:**

Human early growth response-1 (EGR1) is a member of the zing-finger family of transcription factors induced by a range of molecular and environmental stimuli including epidermal growth factor (EGF). In a recently published paper we demonstrated that integrin/EGFR cross-talk was required for Egr1 expression through activation of the Erk1/2 and PI3K/Akt/Forkhead pathways. EGR1 activity and stability can be influenced by many different post-translational modifications such as acetylation, phosphorylation, ubiquitination and the recently discovered sumoylation. The aim of this work was to assess the influence of sumoylation on EGF induced Egr1 expression and/or stability.

**Methods:**

We modulated the expression of proteins involved in the sumoylation process in ECV304 cells by transient transfection and evaluated Egr1 expression in response to EGF treatment at mRNA and protein levels.

**Results:**

We demonstrated that in ECV304 cells Egr1 was transiently induced upon EGF treatment and a fraction of the endogenous protein was sumoylated. Moreover, SUMO-1/Ubc9 over-expression stabilized EGF induced ERK1/2 phosphorylation and increased Egr1 gene transcription. Conversely, in SUMO-1/Ubc9 transfected cells, EGR1 protein levels were strongly reduced. Data obtained from protein expression and ubiquitination analysis, in the presence of the proteasome inhibitor MG132, suggested that upon EGF stimuli EGR1 sumoylation enhanced its turnover, increasing ubiquitination and proteasome mediated degradation.

**Conclusions:**

Here we demonstrate that SUMO-1 modification improving EGR1 ubiquitination is involved in the modulation of its stability upon EGF mediated induction.

## Introduction

Human early growth response-1 (EGR1, also known as NGFI-A, Zif268, Krox24 and Tis8) is the prototypical member of a zinc finger transcription factors family that includes at least three other members (EGR2, -3 and -4) [1], [2]. EGR1 contains a highly conserved DNA-binding domain which recognizes the sequence 5-GCGKGGGCG-3′, a nuclear localization signal, two activator domains and a repressor domain [3]. EGR1 function is negatively regulated by NAB-1 and NAB-2 through specific protein–protein interactions via its repressor domain [4], [5]. Egr1 is induced by a range of molecular and environmental stimuli including growth factors, cytokines, ultraviolet light, ionizing radiation, mechanical injury and fluid biomechanical forces [6]-[11].

EGR1 is involved in the formation of multi-molecular complexes that mediate transcriptional activation or repression of target genes that are involved in the control of cell proliferation and differentiation [12]. Up-regulation of Egr1 expression may result in apparently contradictory activities including mitogenesis, differentiation, tumor suppression, apoptosis, and protection from apoptosis [13], [14]. It was suggested that EGR1 exerts its activity by modulating the expression of different genes involved in different pathways. EGR1 regulates the insulin-like growth factor-II (IGF-II), platelet-derived growth factor-A (PDGF-A) and platelet-derived growth factor-B (PDGF-B) genes, which are known to be involved in cell proliferation [15], [16]; BCL-2, fibronectin, and nuclear factor-B, which are associated with survival and cell differentiation [17], [18]; as well as p53, PTEN, and tumor necrosis factor-alpha (TNFα) which are involved in apoptosis [19]–[21]. Additional relevant targets are VEGF and tissue factor, which are associated with tumor progression, and p57/KIP2 and TGFβ1, which induce growth inhibition in a cell type– dependent manner [22]–[24]. Although its induction is generally transient and greatly dependent on the nature of the various inducers, it appears to be sustained in a high proportion of prostate cancer cell lines and tumors, suggesting that EGR1 stimulates tumor cell growth in certain types of cancer [25], [26]. Indeed, Egr1 over expression promotes growth in several systems, including kidney and endothelial cells. In contrast, in breast, lung, and brain tumors, Egr1 is down-regulated and when re-expressed results in growth suppression and apoptosis [27], [28]. EGR1 is also observed to be required for differentiation and apoptosis in normal and tumor cells; on the basis of these observations, it seems that EGR1 lies at a convergence point and its effects depend on the signals transduced and on the cell context.

**Figure 1:**
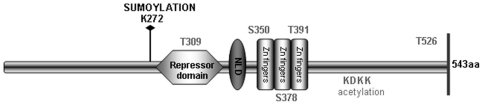
Scheme of EGR1 post-translational modifications.

Moreover different types of post-translational modifications such as phosphorylation, acetylation and ubiquination have been described to regulate the transcriptional activity and the stability of the EGR1 protein (Fig. 1) [29], [30].The acetylation of EGR1, promotes complex formation with p300-CBP that is inhibitory for transcriptional activity of EGR1. It seems that EGR1 acetylation occurs under conditions of low Egr1 induction [31]. In contrast, strong induction of Egr1 by UV irradiations or growth factor stimuli results in a phosphorylation of the induced EGR1 protein [32]. The nature of the post-translational modifications of EGR1 (acetylation or phosphorylation) likely plays a role in the choice of target genes as well as in the effect of EGR1 on the transcription of these genes. More recently, it has been described a novel Akt-Egr-1-alternate reading frame (ARF)-PTEN axis, in which PTEN activation *in vivo* requires ARF-mediated sumoylation of EGR1. This modification is dependent on the phosphorylation of EGR1 at S350 and T309 by Akt, which promotes the interaction of EGR1 with ARF [33]. Sumoylation is a post-translational protein modification leading to the attachment of SUMO (Small-Ubiquitin–like Modifier) to specific lysine residues of target proteins, mainly nuclear proteins. The mechanisms involved in maturation and transfer of SUMO to target substrates are very similar to that seen for ubiquitination and other ubiquitin-like proteins but, differently from ubiquitination, sumoylation alters a number of different functional parameters such as: sub cellular localization, protein partnering, DNA binding and trans-activation functions of transcription factors [34]–[36]. Contrary to the long-standing assumption that SUMO has no role in proteolytic targeting and rather acts as an antagonist of ubiquitin in some cases, it has recently been discovered that sumoylation itself can function as a secondary signal mediating ubiquitin-dependent degradation by the proteasome. Here we show that EGR1 is conjugated to SUMO-1 *in vivo* in response to EGF. We also show that sumoylation enhance EGR1 turnover, increasing its ubiquitination and proteasome mediated degradation.

## Results

### EGF treatment induces Egr1 transient expression in ECV304 cells

**Figure 2:**
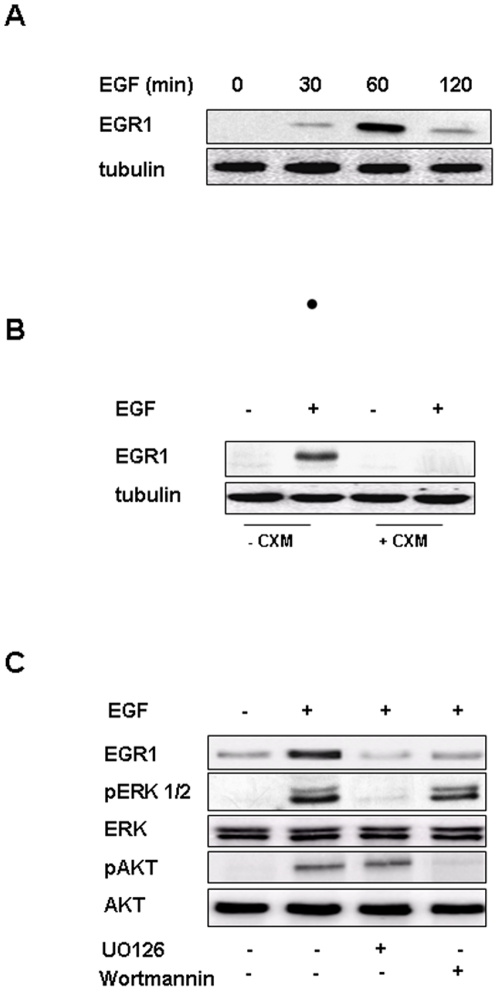
Egr1 is transiently induced in ECV304 cells in response to EGF treatment. (**A**) ECV304 cells were treated for 30, 60 and 120 minutes with 10 ng/ml EGF. Lysates were immunoblotted with polyclonal antibody to EGR1 and, after stripping, with anti tubulin antibody to assess equal loading. (**B**) ECV304 cells were treated for 60 minutes with 10 ng/ml EGF in the absence or in the presence of 20 µM cycloheximide. Lysates were immunoblotted with polyclonal antibody to EGR1 and, after stripping, with anti tubulin antibody to assess equal loading. (**C**) ECV304 cells were treated for 60 minutes with 10 ng/ml EGF in the absence or in the presence of UO126 or wortmannin. Lysates were immunoblotted with polyclonal antibody to EGR1, phospho-ERK 1/2, ERK 1/2, phospho-AKT and AKT. All experiments are representative of three independent.

In a recently published paper we demonstrated that integrin/EGFR cross-talk, through activation of the PI3K/Akt/Forkhead pathway, is required for induction of Egr1 expression in ECV304 cells [37]. Here we show that in adherent cells Egr1 is transiently induced in response to EGF with a peak of induction at 1 hour of treatment (Fig. 2A). Experiments performed in the presence of cycloheximide confirmed that EGR1 is newly synthesized in response to EGF (Fig. 2B). As demonstrated by the use of the specific inhibitors UO126 and wortmannin, both activated ERK 1/2 MAPK and PI3K/Akt signal transduction pathways, are required for EGF induced Egr1 expression (Fig. 2C).

### EGR1 is sumoylated and ubiquitinated in response to EGF

**Figure 3:**
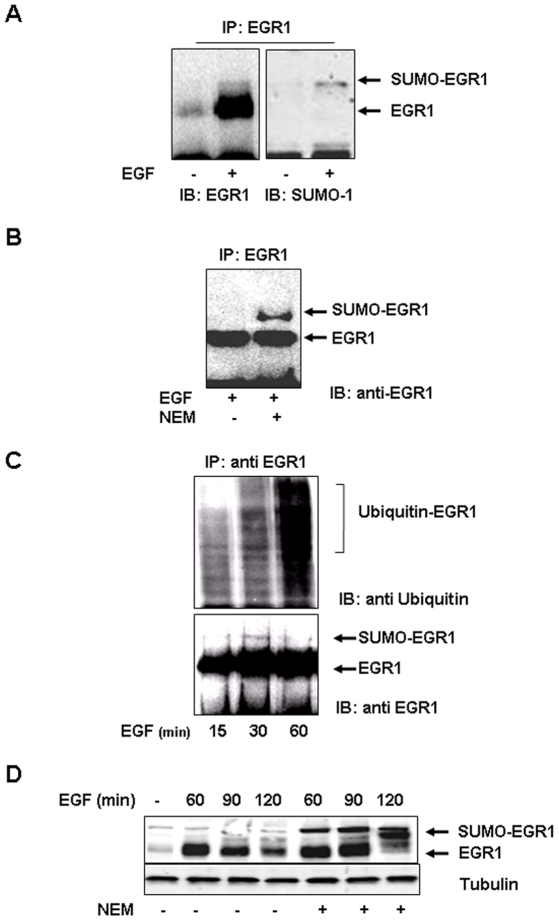
EGR1 induced by EGF is sumoylated and ubiquitinated *in vivo.* (**A**) ECV304 cells were treated for 1 hour with 10 ng/ml EGF to induce Egr1 expression. Lysates were immunoprecipitated with polyclonal antibody to EGR1 and immunoblotted with anti EGR1 and, after stripping, with anti SUMO-1 antibodies. (**B**) ECV304 cells were treated 1 hour with 10 ng/ml EGF in the absence or in the presence of 100 µM NEM and lysates obtained were immunoprecipitated with a polyclonal antibodies to EGR1 and immunoblotted with antibodies anti EGR1. The arrows indicate bands corresponding to EGR1 and EGR1 plus SUMO-1. (**C**) ECV304 cells were treated for 15, 30 or 60 minutes with 10 ng/ml EGF. Lysates were immunoprecipitated with polyclonal antibody to EGR1 and immunoblotted with anti EGR1 and, after stripping, with anti K48 ubiquitin antibodies. (**D**) ECV304 cells were treated for different times with 10 ng/ml EGF in the absence or in the presence of 100 µM NEM and lysates obtained were resolved by SDS-PAGE and analyzed by Western blot with antibodies against EGR1 and tubulin. All experiments are representative of three independent.

It has been recently demonstrated that EGR1 can be sumoylated upon phosphorylation by Akt [32]. In order to verify if EGF treatment induced EGR1 sumoylation *in vivo*, we treated ECV304 cells with EGF for 60 minutes to induce Egr1 expression and then performed immunoprecipitation experiments with EGR1 specific antibodies. Immunoblot analysis with anti EGR1 antibodies evidenced two bands: one corresponding to the endogenous EGR1 (80 kD) and a mild higher-molecular-weight band (90 kD) corresponding to sumoylated EGR1 (Fig. 3A left panel). We confirmed that the higher-molecular-weight band corresponded to sumoylated EGR1 by immunoblot experiments on EGR1 immunoprecipitates with an anti SUMO-1 specific antibody (Fig. 3A right panel). We better evidenced the sumoylated form of EGR1 adding 100 µM *N*-ethylmaleimide (NEM), a specific inhibitor of SUMO proteases, to cell treated 30 minutes with EGF. Again, immunoprecipitation experiments allowed to evidence the sumoylated form of EGR1 (Fig. 3B). Moreover, by immunoblot analysis of EGR1 immunoprecipitates with anti EGR1 and anti-K48 ubiquitin antibodies, we demonstrated that upon 30 minutes of incubation with EGF, EGR1 is prevalently sumoylated, while upon 60 minutes of treatment EGR1 is prevalently poly-ubiquitinated (Fig. 3C). Finally, we demonstrated that sumoylation is a dynamic process that favors EGR1 ubiquitination, in fact, when we treated ECV304 cells for different times with EGF in the absence or in the presence of 100 µM NEM we evidenced by immunoblot analysis that there was a time dependent increase in the sumoylated form of EGR1 without protein degradation (Fig. 3D).

### SUMO1/Ubc9 over-expression affects Egr1 expression

**Figure 4:**
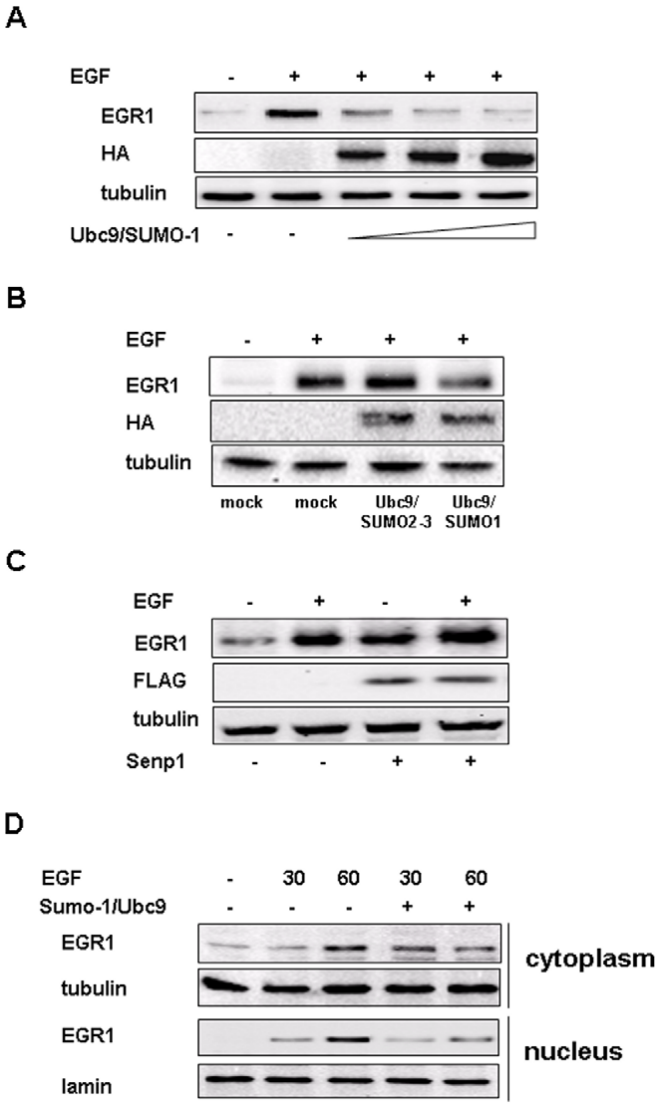
EGF induced Egr1 expression is reduced by SUMO-1 over expression. (**A**) ECV304 cells were transiently transfected with different doses of SUMO-1/Ubc9 expression vectors and, after 24 hours, treated for 1 hour with 10 ng/ml EGF. Cell lysates were subjected to Western blot analysis with anti EGR1 and, after stripping, with anti tubulin antibodies as loading control and anti-HA, as control of transfection. (**B**) ECV304 cells were treated with 10 ng/ml of EGF for 1 hour after 24 hours of transfection with SUMO-1 or SUMO 2/3 and Ubc9 expression vectors. Cell lysates were subjected to Western blot analysis with anti EGR1 antibodies. Western blot with tubulin was used as a loading control and anti-HA as control of transfection. (**C**) ECV304 cells were treated with 10 ng/ml of EGF for 1 hour after 24 hours of transfection with Senp1 expression vector. Cell lysates were subjected to Western blot analysis with anti EGR1 antibody. Western blot with tubulin was used as a loading control and anti-Flag as control of transfection. (**D**) ECV304 cells were transfected with SUMO-1-HA and Ubc9 expression vectors; 24 hours later, cells were treated with EGF 10 ng/ml for 1 hour and processed for nuclear (N)/cytoplasmic (C) fractionation as described in Materials and Methods. A 50-µg sample of each fraction was resolved by SDS-PAGE and analyzed by Western blotting with antibodies against EGR1, lamin as the nuclear marker and tubulin as the cytoplasmic marker. All experiments are representative of three independent.

In order to verify the effects of sumoylation on Egr1 expression we transiently transfected ECV304 cells with different dose of SUMO-1 and Ubc9 expression vectors ranging from 1 to 10 µM and after 1 hour of EGF treatment we performed Western blot analysis. The result shown in Figure 4A evidences that EGF induced EGR1 protein levels decreased proportionally to the amounts of transfected vectors. Moreover, transient transfection with an equal dose of plasmid coding for SUMO2/3 paralogs did not alter EGR1 expression upon EGF treatment, indicating the specificity of SUMO-1 in EGR1 modification (Fig. 4B). Instead, transient transfection with the de-sumoylase Senp1 resulted in an increased expression of basal and EGF induced Egr1 (Fig. 4C). No significant variations in Egr1 expression were observed using a non conjugable form of SUMO-1 lacking gg (data not shown). To investigate whether sumoylation could affect EGR1 localization, we over-expressed SUMO-1 and Ubc9 in ECV304 cells. After 30 or 60 min of EGF stimuli, we lysed cells and isolated the nuclear and the cytosolic fractions. Immunoblot analysis showed that after 60 minutes of EGF treatment, EGR1 protein levels were consistently reduced inside the nucleus while a slight increase was shown at 30 min in the cytoplasm of transfected cells (Fig. 4D).

### SUMO1/Ubc9 over-expression induces Egr1 gene transcription

**Figure 5:**
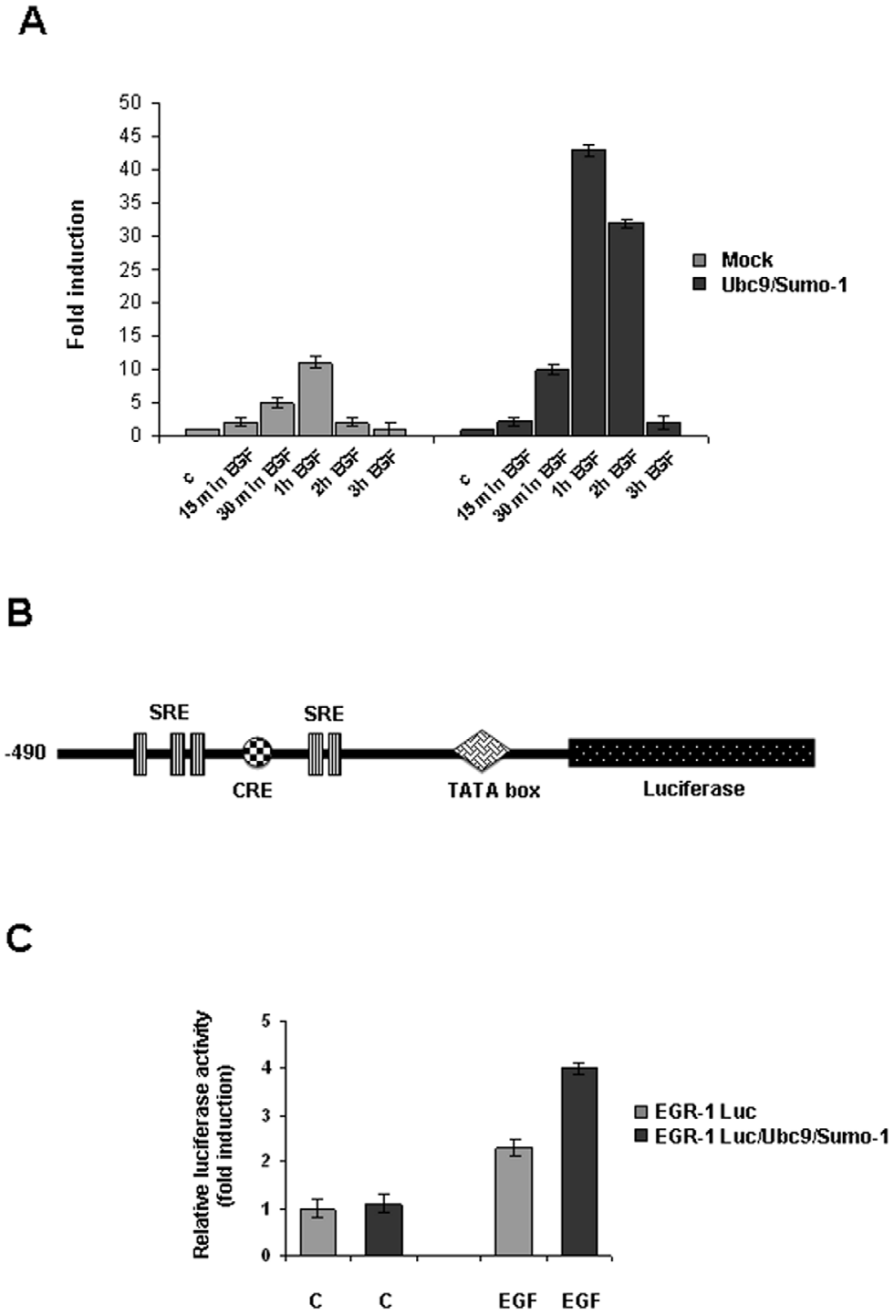
Egr1 gene transcription is transiently induced by SUMO-1 over-expression. (**A**) ECV304 cells were transiently co-transfected with SUMO-1 and Ubc9 expression vectors. 24 hours after transfection, cells were treated with EGF 10 ng/ml for the indicated times to induce Egr1 transcription. Total cellular RNA was extracted and subjected to RT-PCR in which 18s RNA was used as a control. (**B**) An Egr1-luciferase reporter plasmid (Egr luc 1.2 plasmid) was transfected in ECV304 cells together with Ubc9 and SUMO-1 expression vectors, as indicated. A constitutively expressing *Renilla* luciferase (pRL-CMV) plasmid was included as a control of transfection efficiency. Egr1 promoter trans-activation was measured by a dual luciferase assay (*lower panel*). (**C**) Schematic representation of Egr1 promoter. All data are represented as means ± SD of three independent experiments.

In order to verify if the decreased EGR1 protein levels, observed upon SUMO-1/Ubc9 transfection, were due to the modulation of the Egr1 transcription or to an increased degradation of the protein, we measured Egr1 mRNA levels by quantitative RT-PCR. We transiently transfected ECV304 cells with SUMO-1 and Ubc9 expression vectors and analyzed Egr1 mRNA levels at different times of EGF stimuli. Surprisingly, EGF induced Egr1 mRNA levels resulted significantly increased in transfected cells, while the kinetic of induction remained unaltered (Fig. 5A). To confirm these data, we transiently co-transfected ECV304 cells with an Egr1 promoter-luciferase (Fig. 5B), renilla, SUMO-1 and Ubc9 expression vectors. After 8 h of EGF stimulus we measured Egr1 promoter activity by luciferase assay and results indicated that EGF induced Egr1 promoter activity resulted increased in SUMO-1/Ubc9 transfected cells (Fig. 5C).

### SUMO-1/Ubc9 over-expression stabilize EGF induced ERK 1/2 activation

**Figure 6:**
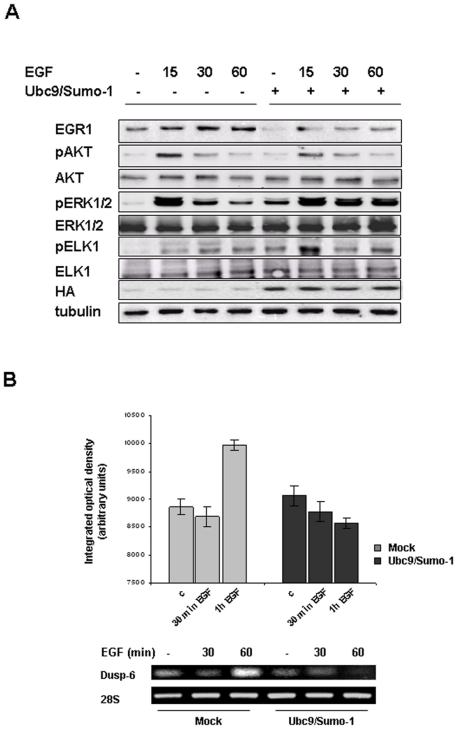
EGF induced ERK1/2 phosphorylation is stabilized by SUMO-1 over expression. (**A**) ECV304 cells transiently transfected with empty vector or SUMO-1-HA and Ubc9 expression vectors were treated for 15, 30 and 60 minutes with 10 ng/ml EGF. Cell extracts were prepared, resolved by SDS-PAGE and immunoblotted with antibodies anti EGR1 phospho-AKT, AKT, phospho-ERK 1/2, ERK 1/2, phospho-ELK1, ELK 1. Filters were probed with anti-tubulin antibody, to verify equal sample loading, and anti-HA antibody, as control of transfection. (**B**) ECV 304 cells transiently transfected with empty vector or SUMO-1-HA and Ubc9 plasmids were treated for 30 and 60 minutes with 10 ng/ml EGF. Total RNA was extracted and a semi-quantitative RT-PCR was performed in order to analyze Dusp6 expression; 28S was used as housekeeping gene. All experiments are representative of three independent.

To investigate if SUMO-1 could modulate the ERK/MAPK or the PI3K/Akt pathways, both involved in the control of Egr1 expression, we transiently transfected ECV304 cells with SUMO-1 and Ubc9 expression vectors, treated cells with EGF for different times and performed Western blot analysis with specific antibodies for phospho-proteins. Results revealed that in transfected cells there was a slight decrease in EGF induced AKT phosphorylation, while ERK 1/2 and consequently Elk1 phosphorylation resulted increased and more stable up to 60 minutes (Fig. 6A). On the basis of these observations, we decided to investigate if stabilization of activated ERK 1/2 could be due to a deficit in DUSP6 phosphatase induction. We performed semi-quantitative RT-PCR experiments in ECV304 cells transiently transfected with SUMO-1 and Ubc9 expression vectors and analyzed Dusp6 mRNA levels at different times of EGF stimuli. As shown in Figure 6B while in control cells Dusp6 expression increased upon 60 minutes of EGF treatment, this increase was not detectable in transfected cells.

### SUMO-1/Ubc9 over-expression enhances EGF induced EGR1 ubiquitination and its proteasome mediated degradation

**Figure 7:**
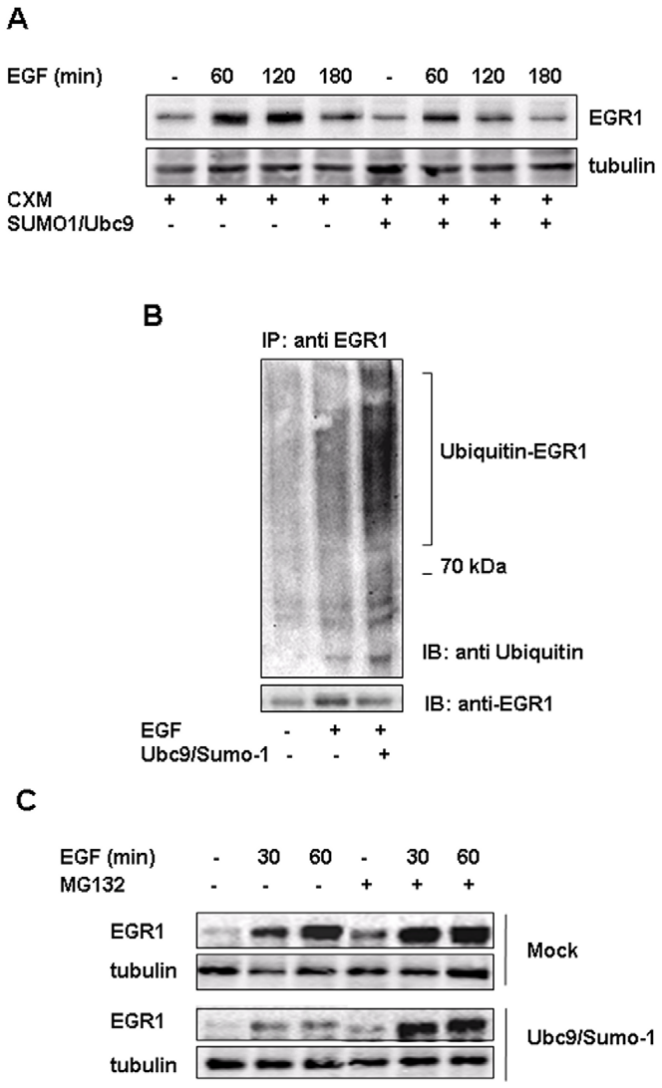
SUMO-1 expression induces EGR1 ubiquitination and proteosome mediated degradation. (**A**) ECV304 cells were transiently co-transfected with SUMO-1 and Ubc9 expression vectors. 24 hours after transfection, cells were treated with EGF 10 ng/ml for the indicated times in the presence of 20 µM cycloheximide. Cell extracts were prepared, resolved by SDS-PAGE and immunoblotted with antibodies anti EGR1 and tubulin for equal loading. (**B**) ECV304 cells were transiently transfected with SUMO-1 and Ubc9 expression vectors and treated for 1 hour with 10 ng/ml EGF. Lysates were immunoprecipitated with polyclonal antibody to EGR1, immunoblotted with anti K48 ubiquitin and, after stripping, with anti EGR1 antibodies. (**C**) ECV304 cells were transfected with SUMO-1/Ubc9 expression vectors,. 24 hours after transfection cells were pre-treated with MG132 (10 µM, lanes 4, 5 and 6) or vehicle (DMSO, lane 1, 2 and 3) for 20 minutes and, then, treated with EGF 10 ng/ml for 30 and 60 minutes to induce Egr1 in the presence of MG132. The protein extracts were analyzed by Western-blot using antibodies against EGR1 or tubulin as loading control. All experiments are representative of three independent.

To investigate the role of sumoylation in the control of EGR1 protein stability, we transiently transfected cells with SUMO-1/Ubc9 expression vectors and in the presence of cycloheximide we treated cells with EGF for different times. Immunoblot analysis evidenced an increased degradation of EGR1 in transfected cells (Fig. 7A). Moreover, in mock or transfected with SUMO-1/Ubc9 expression vectors cells, treated 1 hour with EGF, we performed immunoprecipitation experiments with anti-EGR1 specific antibodies in the presence of the proteasome inhibitor MG132. Immunoblot analysis with anti-Ubiquitin antibodies evidenced that SUMO-1 over-expression enhanced EGR1 ubiquitination upon EGF treatment. These data confirmed that sumoylation increases EGR1 ubiquitination probably enhancing its degradation (Fig. 7B).

In order to verify if sumoylation was involved in the proteasome mediated EGR1 degradation we transiently transfected ECV304 cells with SUMO-1 and Ubc9 expression vectors and treated cells with the specific proteasome inhibitor, MG132. After 30 and 60 minutes of EGF treatment in the absence or in the presence of MG132 we performed Western blot analysis. Results show that transfected cells expressed reduced levels of EGR1 induced by EGF, levels that were increased upon the addiction of MG132. These data suggest that SUMO-1 and Ubc9 promote EGR1 ubiquitination and its proteasome mediated degradation (Fig. 7C).

## Discussion

EGR1 is a short-lived protein, but the mechanism that regulates its stability has not yet been clarified. Modifications that affect Egr1 expression or stability could significantly impact growth factor regulated gene expression and small changes in Egr1 expression levels can have significant impact on cells survival and proliferation of different tumor cell lines.

Previous studies revealed that both the EGR1 DNA binding ability and the transcriptional activity may be influenced by post-translational modifications, such as phosphorylation, glycosylation, and acetylation [29]–[30].

In addiction to these modifications, in a recently published paper it has been described that EGR1 can be sumoylated. Authors indicated that this post-translational modification was dependent on the phosphorylation of EGR1 by AKT, which promoted interaction with ARF and modulated PTEN synthesis creating a negative feedback regulation by PTEN on its own synthesis through PI3 kinase inhibition. Lysine 272 of EGR1 was required for EGR1 sumoylation by the Ubc9/SUMO-1/ARF system [32].

The small ubiquitin-related modifier (SUMO) is a versatile cellular tool to modulate proteins localization and functions. SUMO modification is a reversible process analogous to ubiquitynation. The consecutive actions of E1, E2 and E3 enzymes catalyze the attachment of SUMO to target proteins, while de-conjugation is promoted by SUMO specific proteases. Contrary to the long-standing assumption that SUMO has no role in proteolytic targeting and rather acts as an antagonist of ubiquitin in some cases, it has recently been discovered that sumoylation itself can function as a secondary signal mediating ubiquitin-dependent degradation by the proteasome. The discovery of a novel family of RING finger ubiquitin ligases bearing SUMO interaction motifs implicated the ubiquitin system in the control of SUMO modified proteins. SUMO modification as a signal for degradation is conserved in eukaryotes and ubiquitin ligases that specifically recognize SUMO-modified proteins have been discovered in species ranging from yeasts to humans [36].

In the present work we describe that in ECV304 cells upon EGF stimuli Egr1 is transiently induced and a fraction of endogenous EGR1 is sumoylated, ubiquitinated and, therefore, degraded.

In an attempt to understand the biological relevance of the sumoylation process on Egr1 transcription and/or translation in response to EGF stimuli, we performed experiments in ECV304 cells transiently transfected with proteins involved in this process i.e SUMO1, SUMO 2/3, Ubc9 and Senp1. Data obtained by RT-PCR and luciferase assays in EGF treated ECV304 cells revealed that over-expression of SUMO-1 resulted in a significant up-regulation of Egr1 mRNA levels. We did not deeply characterize the mechanism but we hypotyzed that SUMO-1 over-expression induced an increase and stabilization in ERK 1/2 activity that in turn activate ELK-1 mediated transcription. Moreover, our preliminary data suggest that ERK 1/2 increased phosphorylation could be attributable to a deficit in Dusp6 induction, probably caused by sumoylation dependent repression of Ets-1 transcriptional activity. Moreover a reduction in EGR1 protein levels could abolish a negative feedback control on its own promoter. In fact by chromatin IP experiments that we performed in our cell model (data not shown) we know that upon EGF treatment EGR1 is detached and more pELK-1 is bound to Egr1 promoter. Moreover, it must be considered that the activity of different transcription factors that bind Egr1 promoter, such as NCoR, p300, CREB and ELK-1, is modulated by sumoylation.

It is well known that upon serum and EGF stimuli EGR1 localize into the nucleus where it is involved in the regulation of gene expression. Although sumoylation can alter intracellular localization of target proteins, we found a general reduction but not a significant change in EGR1 localization in cells that over-expressed SUMO-1; in fact, in contrast with mRNA expression data, western blot analysis showed that in ECV304 over-expressing SUMO-1 cells EGR1 protein levels were significantly reduced. A recent study reports, by yeast two-hybrid screening, that EGR1 binds C8 proteasome subunit and that EGR1 protein is targeted for proteolysis by the ubiquitin-dependent proteasome pathway. In order to verify if sumoylation is involved in the proteasome mediated EGR1 degradation we performed Western blot analysis in ECV304 cells in the presence of the specific proteasome inhibitor MG-132. We observed that in SUMO-1 over-expressing cells, EGF induced EGR1 protein levels returned to be comparable to the controls in the presence of MG-132.

In this study, several lines of evidence support the notion that sumoylation of EGR1 might be involved in the protein stability, into the control of its turnover and in its proteasome-mediated degradation.

## Materials and Methods

### Reagents and antibodies

Polyclonal antibodies to EGR1 and ERK 1/2 were obtained from Santa Cruz Biotechnology (Santa Cruz, CA). Monoclonal antibodies to AKT and polyclonal antibodies to phospho-p42/p44 ERK (Thr202/Tyr204) were purchased from Upstate Biotech. Monoclonal antibodies to SUMO-1 and Ubiquitin were purchased from Zymed (San Francisco, CA). Monoclonal antibodies to Lamin and Tubulin, anti FLAG, conjugated secondary antibodies human, recombinant EGF, wortmannin, UO126 and MG-132 were obtained from Sigma-Aldrich (St. Louis, MO). Monoclonal antibody to HA epitope was purchased from Roche Applied Science. Protein A-Sepharose, PVDF and nitrocellulose membranes were from Amersham Biosciences (Piscataway, NJ); ECL reagents were from Biorad. Culture media, sera and the Lipofectamine2000 reagent were from Invitrogen (Carlsbad, CA).

### Plasmids

Plasmids pHA-Sumo-1, pHA-Sumo-1-Δ6, and pHA-Ubc9 were kindly provided by Dott. L. Collavin, Trieste University, Italy. Plasmid pLUC-Egr-1,1.2 was kindly provided from Prof. G. Thiel, Homburg University, Germany. Flag-Senp-1 and Sra-HA-Sumo-2 plasmids were from Addgene.

### Cell culture and transfection

Human ECV304 endothelial cell line was purchased from ATCC (ATCC® CRL-1998) and grown in Dulbecco's modified Eagle's medium (DMEM) supplemented with 10% heat-inactivated fetal calf serum (FCS) and penicillin/streptomycin (100 U/ml and 100 µg/ml, respectively) at 37°C in a 5% CO_2_ atmosphere. Before treatments with EGF cells were starved over night in serum free medium. ECV304 cells grown to 80% confluence in 100 mm tissue culture dishes were transiently transfected by the Lipofectamine2000 reagent as described by the manufacturer.

### Cell lysis, immunoprecipitation and Western blotting

Cells were extracted with RIPA buffer (0.5% Triton x-100, 0.5% NP40, 150 mM NaCl, 50 mM Tris-HCl pH 7.8, 5 mM EDTA, 0.4 mM Na_3_VO_4_, 0.1% sodium deoxycholate, 20 mM NEM, 10 µg/ml leupeptin, 4 µg/ml pepstatin and 0.1 unit/ml aprotinin). Cell lysates were centrifuged at 13.000 x *g* for 10 minutes and the supernatants were collected and assayed for protein concentration with the Bio-Rad protein assay method (Bio-Rad, Hercules, CA). Proteins were run on SDS-PAGE under reducing conditions. Cell fractionation was performed according the protocol described by Scheiber et al. [38]. For immunoprecipitation experiments, proteins were incubated with the appropriate antibody for 1 hour at 4°C as previously described [39] in the presence of 30 µl protein A-Sepharose beads. Protein A-Sepharose beads were then added to 3 mg of protein cell extract to collect immunoprecipitates. The beads were washed three times with 1 ml of Tris buffered saline, 0.5% Triton X-100 and once with 1 ml of Tris-buffered saline, 0.5% Triton X-100, 0.1% SDS and the immunoprecipitates were eluted by boiling the beads in 2X Laemmli sample buffer for 5 minutes. The immunoprecipitates were resolved on 6% or 8% SDS-polyacrylamide gel and transferred onto nitrocellulose for immunoblotting, reacted with specific antibodies, and then detected with peroxidase-conjugated secondary antibodies and enhanced chemiluminescence reagents. When appropriate, the nitrocellulose membranes were stripped according to manufacturer's recommendations and reprobed. Densitometric analysis was performed using the GS 250 Molecular Imager (Bio-Rad). For ubiquitin detection, proteins were transferred on a PVDF (polyvinylidene fluoride) membrane, previously activated by incubation in 100% methanol for 5 minutes at room temperature. After transfer, filters were subjected to a treatment in denaturing solution for 30 minutes at 4°C (6 M guanidium chloride, 20 mM Tris pH 7.5, 1 mM PMSF and 5 mM β-mercaptoethanol). After extensive washing in TBS-T buffer (TBS Tween 0,1%), filters were blocked overnight at 4°C in 5% BSA (dissolved in TBS-T). After blocking, filters are incubated with the P4D1 antibody (Santa Cruz, 1∶1000) or FK2 (Biomol, 1∶1000) against Ub, for 1 hour at room temperature, then with the anti-mouse horseradish peroxidase-conjugated secondary antibody revealed using the Enhanced Chemiluminescence method.

### RNA isolation and analysis of nucleic acid level by quantitative PCR

Total RNA was isolated by the guanidinium thyocianate method (as described in [40]. The cDNA used as template for amplification in the Real Time quantitative PCR was constructed by reverse transcription reaction using SuperScript II (Invitrogen), with random hexamers as primers, starting with equal amounts of RNA. As a PCR internal control, 18S rRNA was simultaneously amplified using the primers: 5′-CCCACTCGG CACCTTACG-3′ (forward) and 5′-TTTCAGCCTTGCGACCATACT-3′ (reverse). The primer sequences for the Egr-1 gene were as follows: 5′-CCTGCGACATCTGTGGAAGAA-3′ (forward) and 5′-CGCAAGTGGATCTTGGTATGC-3′ (reverse). Quantitative Real Time PCR was performed using double stranded DNA binding dye SYBR Green PCR Master mix (Applied Biosystems) in an ABI GeneAmp 7000 Sequence Detection System. Each reaction was run in triplicate and the melting curves were constructed, using Dissociation Curves Software (Applied Biosystems) to ensure that only a single product was amplified. As Real Time quantitative PCR control 18S rRNA was also analyzed. Semi quantitative competitive reverse transcription chain reaction RT-PCR for Dusp6 was performed according published indications [41] using as specific primers for Dusp6 5′-GTTTTTCCCTGAGGCCATTT-3′ (forward) and 5′-TAGGCATCGTTCATCGACAG-3′ (reverse). A ratio between the level of expression of the target gene and that of the house-keeping gene 28S in total RNA samples was determined. Fold induction of the target gene was calculated by ascribing the ratio as 1 for control-treated samples. All results of RT-PCR were analyzed from three PCR results.

### Luciferase Assays

For luciferase assays, ECV304 cells in 3-cm Petri dishes were lipofected with 400 ng of the reporter, 250 ng of Egr1 promoter plasmid [42], and 500 or 400 ng of SUMO-1 and Ubc9. In all samples, 40 ng of the plasmid pRL-CMV (Promega, Fitchburg, WI) encoding Renilla luciferase were included for normalization of transfection efficiency. After 36 hours, cells were lysed and assayed by using the Dual Luciferase kit (Promega). Relative luciferase activity is the ratio of firefly to Renilla luciferase activity, normalized to the activity of the reporter alone. Expression levels of transfected proteins were verified by immunoblotting of the lysates normalized for transfection efficiency.
